# Prostatic solitary fibrous tumor masquerading as prostate cancer on multiparametric MRI: A case report

**DOI:** 10.1016/j.eucr.2026.103666

**Published:** 2026-11-05

**Authors:** Farhood Khaleghi Mehr, Farshad Banouei

**Affiliations:** Department of Urology, Hasheminejad Kidney Center, School of Medicine, Iran University of Medical Sciences, Tehran, Iran

## Abstract

Prostatic solitary fibrous tumor (SFT) is exceptionally uncommon and can be mistaken for aggressive prostate cancer on imaging. A 59-year-old man presented with obstructive urinary symptoms and a normal serum PSA. Multiparametric MRI showed a PI-RADS 5 nodule breaching the prostatic capsule. Biopsy revealed bland spindle cells expressing STAT6, CD34, CD99, and BCL-2, with a Ki-67 index of 1%, establishing the diagnosis. After multidisciplinary review and counseling, he elected surveillance instead of resection; MRI at 12 months showed no progression. Tissue confirmation should precede radical treatment of such lesions.

## Highlights

• Prostatic solitary fibrous tumors (SFTs) are very uncommon and may radiographically resemble clinically significant prostatic carcinoma.
• Tissue diagnosis with STAT6 and CD34 immunohistochemistry before radical treatment of a PI-RADS 5 lesion with extracapsular extension and normal PSA averted radical therapy for presumed prostate cancer.
• CD34 and STAT6 immunohistochemistry are essential for diagnosis, as imaging cannot differentiate between solitary fibrous tumors (SFT) and prostate carcinoma.
• Since SFT does not secrete PSA, the standard PSA test is not useful for monitoring tumor growth.
• After tumor board discussion and detailed counseling, the patient chose to defer resection; surveillance with predefined triggers for resection showed no progression at one year, but it cannot yet be considered a validated management strategy.

## 1 Introduction

Solitary fibrous tumor (SFT) is a rare mesenchymal tumor that typically originates in the pleura in its classic form,^1^ with an incidence rate ranging from 0.37 to 0.61 per million.^2^ Approximately 30 percent of SFTs arise in extrathoracic regions and are characterized by a specific genetic alteration known as the NAB2-STAT6 gene fusion.^1^

Prostatic solitary fibrous tumor (SFT) is extremely rare. Previous reviews have reported approximately 25 to 39 cases,^1^, ^3^ but the most recent systematic review documented a total of 74 cases.^4^ Most cases of prostatic SFT present with irritative urinary symptoms or urinary retention^3^, ^5^ and closely resemble benign prostatic hyperplasia (BPH), as imaging features are nonspecific.^2^ Many patients initially underwent transurethral resection of the prostate (TURP) for suspected BPH, with subsequent pathological analysis revealing SFT.^3^

The diagnosis is based on the nuclear expression of the STAT6 protein, along with the CD34 and Bcl-2 markers. Prostatic solitary fibrous tumors (SFTs) generally follow a benign course; however, aggressive behavior can occasionally occur. The recommended treatment is complete surgical excision with clear margins, as adjuvant therapy is typically not indicated.^2^ Although MRI mimicry of clinically significant cancer by prostatic SFT has been described,^2^, ^4^, ^6^ the instructive elements of this case are the decisions that followed. We describe a 59-year-old man with a PI-RADS 5 lesion with extracapsular extension and a normal PSA level, in whom tissue diagnosis with STAT6 immunohistochemistry preceded any radical treatment and who, after multidisciplinary discussion and counseling, chose to defer resection.

## 2 Case presentation

A 59-year-old man reported experiencing irritative and obstructive lower urinary tract symptoms (LUTS) for three months, with an International Prostate Symptom Score (IPSS) of approximately 19. His medical history was unremarkable, with no family history of malignancy, he was a nonsmoker, and he was not taking any regular medications. Digital rectal examination revealed a soft prostate gland with a palpable nodule in the right lobe. Transrectal ultrasonography showed a prostate volume of approximately 36 mL and a nodule measuring 19 × 24 mm in the right peripheral zone, consistent with the findings from the digital rectal examination. The MRI-derived prostate volume (48.5 cm^3^, below) was taken as the reference value; the smaller TRUS estimate was attributed to operator dependence and the limitations of ultrasound volumetry. Serum PSA was within the normal range (0.22 ng/mL), corresponding to a PSA density of approximately 0.005 ng/mL/cm^3^, calculated with the MRI volume.

Multiparametric MRI was performed on a 1.5 T magnet using a pelvic phased-array surface coil, following the standard technical recommendations outlined in the PI-RADS v2.1 guidelines. The protocol included axial, coronal, and sagittal T2-weighted imaging; diffusion-weighted imaging with a high b-value (≥1400 s/mm^2^) and corresponding apparent diffusion coefficient (ADC) mapping; and dynamic contrast-enhanced (DCE) sequences. The examination revealed a prostate gland measuring 49 × 44 × 43 mm (total volume approximately 48.5 cm^3^) with a distinct T2 hypointense nodule in the right peripheral zone, corresponding to the location identified by digital rectal examination and transrectal ultrasonography. The nodule exhibited marked diffusion restriction, intense early enhancement, and exophytic extension through the capsule into the right anterolateral periprostatic tissue, with a maximal diameter of 26 mm (Fig. 1A and B). It was assigned a PI-RADS v2.1 score of 5. Additional proton MR spectroscopic imaging (multivoxel two-dimensional chemical shift imaging technique) was also performed (Fig. 1C), revealing a dominant citrate peak (relative integral ratio of 1.32), only slightly elevated choline (0.253), and low creatine (0.0811) levels, resulting in a very low (choline + creatine)/citrate ratio, which is uncommon for aggressive cancer. However, extracapsular extension was detected on conventional anatomic MRI images, as spectroscopy cannot reveal this feature (Fig. 1).

**Fig. 1:**
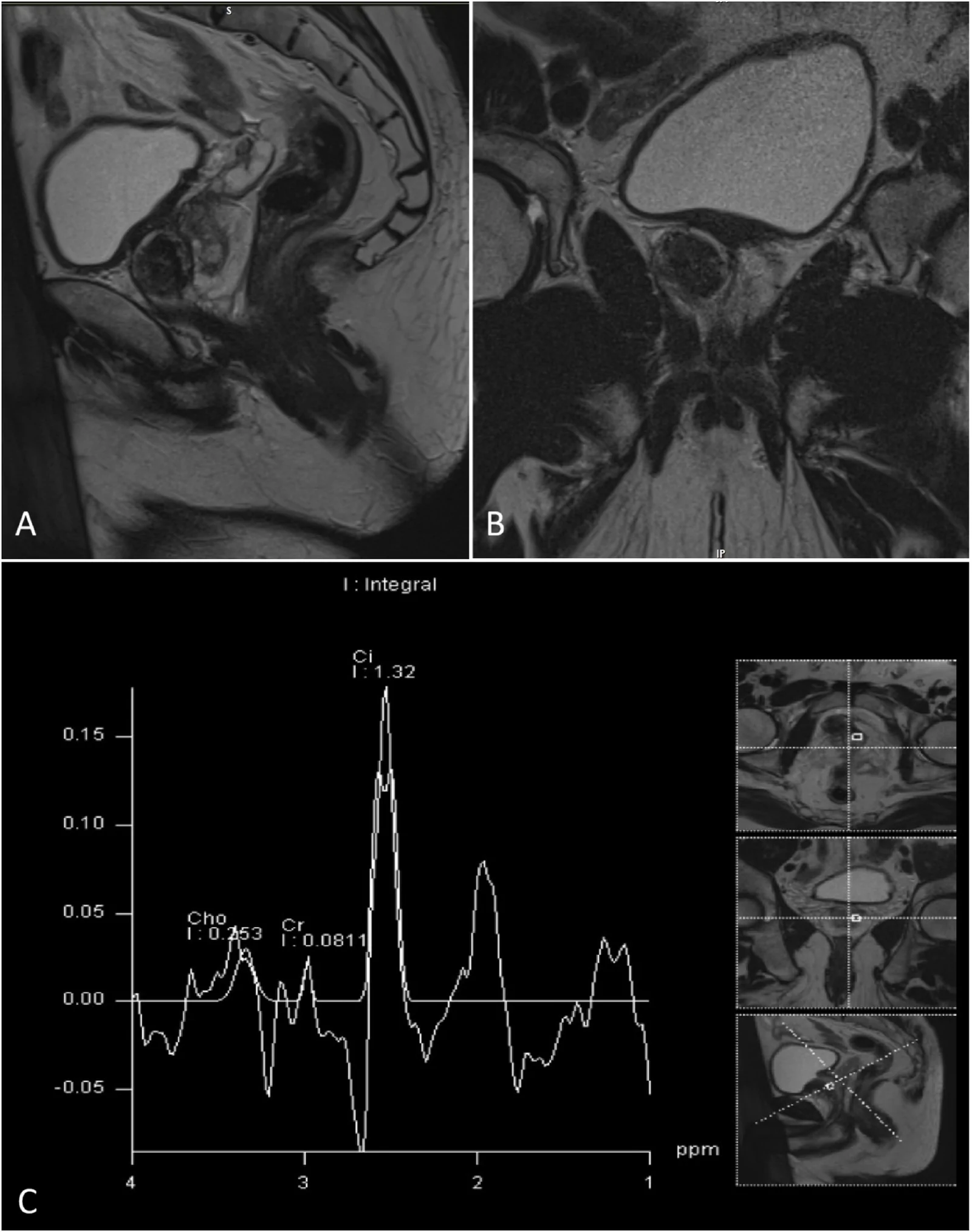
A & B. MRI shows the nodule in the right peripheral zone with exophytic extension beyond the capsule. C. Magnetic resonance spectroscopy (MRS) shows a dominant citrate peak with only mildly elevated choline, a pattern atypical for aggressive malignancy despite the PI-RADS 5 category assigned on conventional sequences (Figure submitted as a separate file: Fig. 1 jpeg).

Subsequently, an MRI-guided targeted biopsy was performed. A 12-core systematic biopsy was also performed, and its findings were concordant with those of the targeted cores. Microscopic examination revealed cells that were ovoid to spindle-shaped with poorly defined cell borders, arranged in a haphazard or short fascicular pattern within a hyalinized to collagenous stroma, exhibiting streaming between collagen fibers and staghorn-shaped blood vessels. The mitotic index was low (1/10 high-power fields), with no evidence of atypia or necrosis. Immunohistochemistry results showed positivity for CD34 (clone QBEnd/10) and STAT6 (clone YE361), focal positivity for PR (clone PgR636), and a Ki-67 (clone MIB-1) proliferation index of 1% (Fig. 2). Markers including C-kit/CD117 (polyclonal), DOG1 (clone K9), SMA (clone 1A4), desmin (clone D33), caldesmon (clone h-CD), and S100 (polyclonal) were negative. Staining for CD99 and BCL-2, performed subsequently on the paraffin block, was also positive, completing the classic CD34/CD99/BCL-2 profile. No molecular testing (e.g., for the NAB2::STAT6 fusion) was performed.

**Fig. 2:**
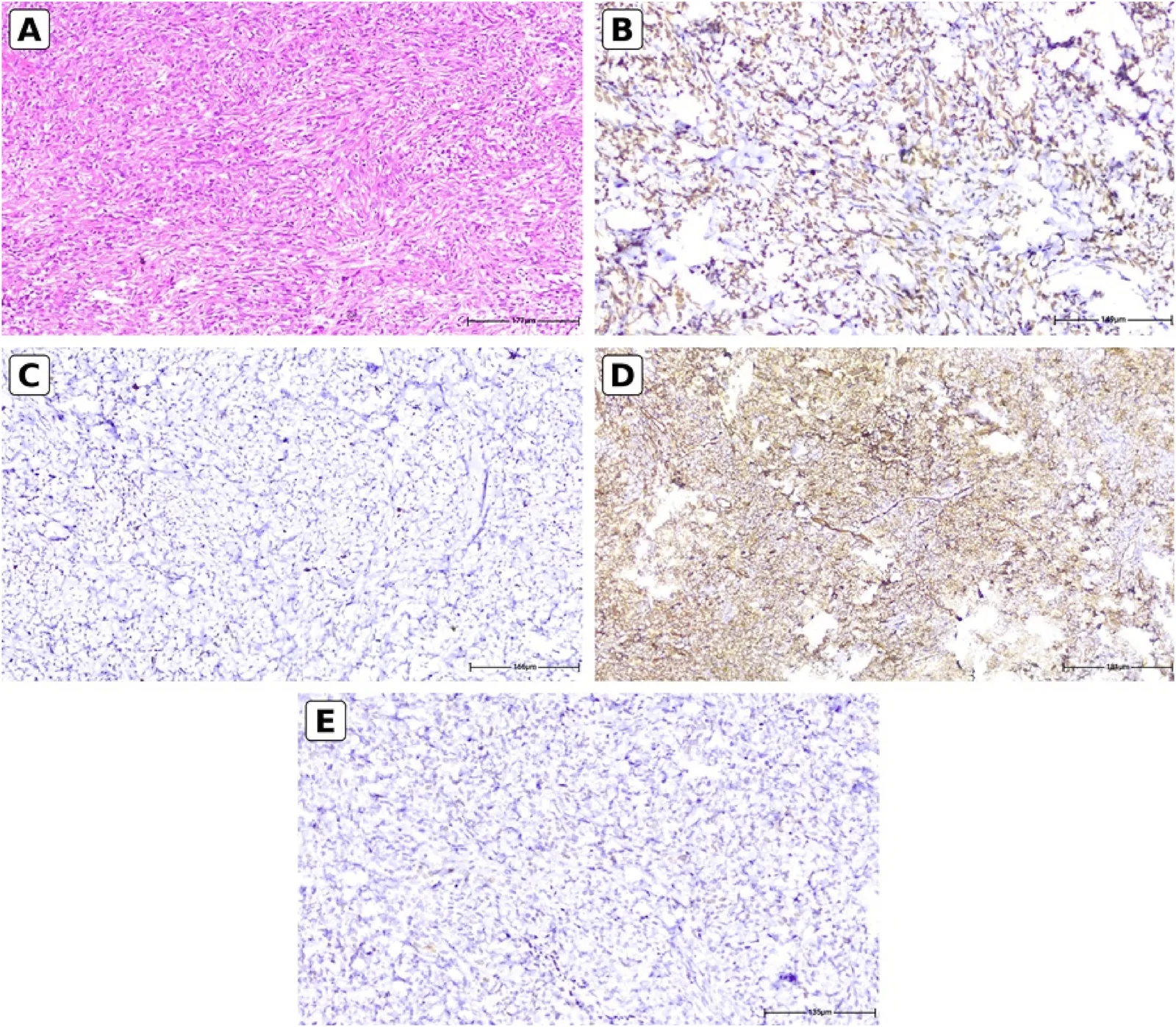
Histopathology of the targeted biopsy (scale bar shown in each panel). (A) Hematoxylin and eosin (H&E)–stained section showing ovoid to fusiform spindle cells with indistinct borders arranged haphazardly or in short, ill-defined fascicles. (B) STAT6 immunostaining (clone YE361) showing nuclear positivity in spindle cells. (C) Ki-67 immunostaining (clone MIB-1) showing a very low proliferative index, with nuclear positivity in only 1% of spindle cells. (D) CD34 immunostaining (clone QBEnd/10) showing cytoplasmic positivity in spindle cells. (E) Progesterone receptor immunostaining (clone PgR636) showing nuclear positivity in a few spindle cells. Negative markers (not shown): C-kit/CD117 (polyclonal), DOG1 (clone K9), smooth muscle actin (clone 1A4), desmin (clone D33), caldesmon (clone h-CD), and S100 (polyclonal) (Figure submitted as a separate file: Fig. 2 png).

Because of the favorable histological features—low mitotic activity, absence of necrosis and atypia, and a Ki-67 index of 1%—despite the PI-RADS 5 findings, the case was presented to a multidisciplinary tumor board of urologic oncologists, a radiologist, a hematologist-oncologist, and other related specialists. Opinions were divided: some members favored resection and others surveillance. The patient received a full explanation of the risks, benefits, and morbidity of surgery and of the risks of surveillance, and the final decision was left to him; he chose to defer surgery for the time being. Resection was to be reconsidered for measurable growth on MRI, involvement of adjacent structures, worsening LUTS or urinary retention, a change in imaging features, or patient preference. Follow-up consisted of clinical visits every three months and annual MRI, with PSA monitored primarily to rule out prostatic adenocarcinoma, since SFTs do not secrete PSA. A repeat multiparametric MRI after 12 months showed no signs of progression. After one year, symptoms had improved (IPSS from approximately 19 to approximately 9), PSA remained normal and stable, and there were no signs of disease progression. The follow-up is ongoing.

Written informed consent to publish was obtained from the patient.

## 3 Discussion

Mimicry of clinically significant prostate cancer by prostatic SFT on MRI is already described.^2^, ^4^, ^6^ The instructive elements here are therefore two decisions^1^: obtaining tissue, with STAT6 immunohistochemistry, before radical treatment of a PI-RADS 5 lesion with extracapsular extension and a normal PSA level; and^2^ the subsequent decision, taken jointly with the patient, not to excise the tumor immediately.

SFT was first reported to occur in the pleura in 1931. Its occurrence in prostatic tissue is exceedingly rare, with fewer than 80 cases documented, primarily as individual case reports.^1^, ^3^, ^4^, ^6^, ^7^, ^8^, ^9^, ^10^ This rarity may be attributed to underdiagnosis, given the similarity of prostatic SFT to benign prostatic hyperplasia (BPH) and carcinoma.

Typically, patients are between their fifth and seventh decades of life and present with either irritative or obstructive symptoms, along with a normal PSA level. As in this case, some reported instances have initially been misdiagnosed as benign prostatic hyperplasia (BPH).^3^, ^5^, ^6^, ^8^ Radiographic findings are generally unremarkable; solitary fibrous tumors (SFT) may show low PI-RADS scores or, as in this case, exhibit prominent diffusion restriction, avidity, and extracapsular extension—features that resemble clinically significant prostate cancer (PI-RADS 5).^2^, ^4^, ^6^

Under the microscope, solitary fibrous tumors (SFT) display bland spindle cells accompanied by staghorn-shaped blood vessels. Due to the presence of overlapping spindle cell tumors in the prostate gland—including STUMP, stromal sarcoma, leiomyoma, and sarcomatoid carcinoma—immunohistochemical staining is crucial for accurate diagnosis.^11^, ^12^ The NAB2-STAT6 gene fusion is a hallmark of SFT, with nuclear STAT6 expression serving as a reliable surrogate marker.^13^, ^14^ In this case, the tumor tested positive for STAT6 and CD34 (and, on additional staining, CD99 and BCL-2, completing the classic CD34/CD99/BCL-2 profile) but negative for SMA, desmin, caldesmon, and other neural/GIST-related markers. No molecular testing beyond immunohistochemistry was performed, so the NAB2::STAT6 fusion itself was not demonstrated.

Solitary fibrous tumors (SFTs) are generally benign in most cases; however, predicting their behavior based solely on morphology can be challenging. The Demicco risk stratification system incorporates factors such as age, tumor size, mitotic activity, and necrosis to estimate the risk of metastasis.^15^ According to this system, based on the biopsy sample, the SFT was classified as low-risk due to low mitotic activity and the absence of necrosis. Nevertheless, mitotic activity and necrosis were assessed only on the core biopsy sample, not the entire tumor, which may lead to underestimation of these features in other tumor regions. Therefore, biopsy-based risk stratification should be interpreted with caution and cannot replace the risk assessment performed on the complete excision specimen.

Surgical removal with negative margins remains the gold standard for treating prostatic solitary fibrous tumors (SFT) and is considered curative, with no indication for adjuvant therapy in low-risk, completely resected cases.^2^, ^4^, ^6^, ^7^, ^8^, ^16^ Deferring resection was therefore a deliberate departure from the standard. It rested on the favorable biopsy features, a tumor board discussion in which opinions were divided, thorough counseling about both options, and, ultimately, the patient's own choice. Surveillance was not open-ended: resection would be reconsidered for measurable growth on MRI, involvement of adjacent structures, worsening LUTS or urinary retention, a change in imaging features, or patient preference. Prostate-specific antigen (PSA) levels were also monitored, primarily to exclude concomitant prostatic adenocarcinoma; since SFTs do not secrete PSA, stable normal PSA levels do not provide information about potential SFT growth or recurrence. Surveillance of SFT requires regular clinical evaluations and scheduled imaging studies; our patient was seen every three months, with MRI performed annually. After one year of follow-up, his symptoms had improved (IPSS from approximately 19 to approximately 9), PSA remained normal and stable, and the follow-up MRI showed no signs of disease progression. He is currently enrolled in a regular follow-up program. Although one-year active surveillance in a single patient is feasible, it cannot be considered definitively safe, and further data are needed.

Natural history and follow-up interval. Reliable data on the growth rate of prostatic SFT are lacking, and no validated surveillance protocol exists. SFTs are generally described as slow-growing, but behavior can be unpredictable: in a previously reported prostatic SFT under surveillance, tumor diameter increased by approximately 2 cm over three years,^6^ and a pleural SFT that had been stable for three years later grew from 8 to 15 cm over five years.^17^ Because progressive growth may worsen LUTS and compromise the chance of complete resection, we combined three-monthly clinical review with annual MRI and the resection triggers listed above; given this uncertainty, shorter imaging intervals during the first years of surveillance may be reasonable.

Limitations: This study has several limitations. First, as it involves a single case, the results cannot be generalized, and the true prevalence of this type of discrepancy in prostatic solitary fibrous tumor (SFT) remains unknown. Second, the risk stratification, which was based on mitotic activity and necrosis, relied on a limited biopsy specimen rather than the entire excised tumor. Third, the diagnosis rests on morphology and immunohistochemistry; the NAB2::STAT6 fusion was not tested molecularly. Fourth, one year of follow-up is too short to judge the safety of deferring resection or to define the growth rate of prostatic SFT.

## 4 Conclusion

This case highlights that prostatic solitary fibrous tumor (SFT) can closely mimic clinically significant prostate cancer, which makes tissue diagnosis, including STAT6 and CD34 immunohistochemistry, an essential step before radical treatment of a PI-RADS 5 lesion with normal PSA. Deferring resection was reasonable here because of favorable biopsy features, multidisciplinary discussion, an informed patient choice, and predefined triggers for resection. It supports maintaining a low threshold for performing STAT6 and CD34 immunohistochemistry in prostatic spindle cell lesions, along with long-term, imaging-based follow-up regardless of the chosen management strategy. Given the one-year follow-up in this single patient, who remains on a structured surveillance plan, active surveillance should be considered an approach attempted in this case rather than a validated or generalizable management recommendation for prostatic SFT.

## CRediT authorship contribution statement

**Farhood Khaleghi Mehr:** Investigation. **Farshad Banouei:** Writing – review & editing, Writing – original draft, Visualization, Validation, Supervision, Software, Resources, Project administration, Methodology, Funding acquisition, Formal analysis, Data curation, Conceptualization.

## Consent for publication

Written informed consent for publication of this case report and any accompanying images was obtained from the patient.

## Ethics declaration

The author(s) declare(s) that the study was approved by the Research Ethics Committee of the School of Medicine, Iran University of Medical Sciences, Tehran, Iran (approval code: IR.IUMS.FMD.REC.1405.386).

Written informed consent for publication of this case report and any accompanying images was obtained from the patient.

## Declaration of generative AI and AI-assisted technologies in the writing process

During the preparation of this work, the author(s) used Claude (Anthropic) to assist with editing and revising the manuscript and the response to the reviewers. After using this tool, the author(s) reviewed and edited the content as needed and take(s) full responsibility for the content of the publication.

## Funding

The author(s) declare(s) that no financial support was received for the research, authorship, or publication of this article.

## Declaration of competing interest

The author(s) declare(s) that they have no known competing financial interests or personal relationships that could have appeared to influence the work reported in this paper.
